# Glial phagocytic clearance in Parkinson’s disease

**DOI:** 10.1186/s13024-019-0314-8

**Published:** 2019-04-05

**Authors:** Marie-Eve Tremblay, Mark R. Cookson, Laura Civiero

**Affiliations:** 1 Axe Neurosciences, Centre de Recherche du CHU de Québec, Université Laval, Quebec, QC Canada; 2 Département de Médecine Moléculaire, Faculté de Médecine, Université Laval, Quebec, QC Canada; 3 Laboratory of Neurogenetics, National Institute on Aging, National Institutes of Health, Bethesda, MD USA; 4 Department of Biology, University of Padova, Via Ugo Bassi 58/B, 35131 Padova, Italy

**Keywords:** Reactive astrocytes, Reactive microglia, Phagocytosis, Parkinson’s disease

## Abstract

An emerging picture suggests that glial cells’ loss of beneficial roles or gain of toxic functions can contribute to neurodegenerative conditions. Among glial cells, microglia and astrocytes have been shown to play phagocytic roles by engulfing synapses, apoptotic cells, cell debris, and released toxic proteins. As pathogenic protein accumulation is a key feature in Parkinson’s disease (PD), compromised phagocytic clearance might participate in PD pathogenesis. In contrast, enhanced, uncontrolled and potentially toxic glial clearance capacity could contribute to synaptic degeneration. Here, we summarize the current knowledge of the molecular mechanisms underlying microglial and astrocytic phagocytosis, focusing on the possible implication of phagocytic dysfunction in neuronal degeneration. Several endo-lysosomal proteins displaying genetic variants in PD are highly expressed by microglia and astrocytes. We also present the evidence that lysosomal defects can affect phagocytic clearance and discuss the therapeutic relevance of restoring or enhancing lysosomal function in PD.

## Background

Parkinson’s disease (PD) is a neurodegenerative condition characterized as a movement disorder of bradykinesia, tremor, rigidity, and postural instability, accompanying a number of non-motor symptoms [1]. Several etiological factors have been identified that contribute to the lifetime risk of PD, including a strong effect of aging [2]. However, numerous studies have confirmed that genetic factors contribute to the pathogenesis of PD. Highly penetrant mutations producing rare, monogenic forms of the disease were discovered in several genes and unique variants with incomplete penetrance were shown to be strong risk factors for PD. Moreover, more than 20 variants are now recognized to modulate the risk for apparently sporadic PD [3] and the largest-to-date meta-analysis revealed over 100 semi-independent risk variants (BioRxiv, 10.1101/388165). Therefore, the risk of developing PD is determined by both non-genetic and genetic factors.

The clinical symptoms of PD reflect the underlying systemic neurodegeneration and protein deposition. A common denominator of both inherited and sporadic forms of PD is the loss of dopaminergic (DA) neurons of the *substantia nigra pars compacta* projecting to the putamen that control voluntary movements [4]. Additionally, proteinaceous inclusions mainly composed by the protein α-synuclein (α-syn) are located in the perikarya (Lewy Bodies, LBs) and within the cell processes (Lewy neurites, LNs) of the surviving nerve cells. Using protein deposition pathology as a marker of disease progression, it was shown that the *substantia nigra* is affected later over the course of pathology compared with other regions of the nervous system, including from the autonomic and enteric nervous system, while it precedes the extensive cortical pathology [5]. Therefore, PD is a multi-system and complex disorder where neuronal dysfunction evolves over time.

Although less often discussed than neuronal pathology, α-syn-containing inclusions in astrocytes have been repeatedly detected in the *substantia nigra*, cerebral cortex and other brain regions in idiopathic PD samples [6, 7]. The density of α-syn immunoreactive astrocytes parallels the occurrence of LNs and LBs in neurons [6]. Neuronal loss and the presence of cytoplasmic inclusions in neuronal and non-neuronal cells are also accompanied by reactive changes of astrocytes and microglia referred to as gliosis. Microglia as well as astrocytes are inflammatory cells that express immune-associated molecules including the major histocompatibility complex (MHC) class II, pro-inflammatory cytokines, and inducible oxide synthase (iNOS). Moreover, astrocytes become hypertrophic and accumulate the intermediate filament protein, glial fibrillary acidic protein (GFAP) [4, 8].

Although reactive glial cells and the upregulation of cytokines was found in the brains and cerebrospinal fluid of patients with PD, the role of neuroinflammation in the pathogenesis of PD is still undetermined [2]. Neuroinflammation in PD has long been considered a downstream response to neuronal damage. However, alteration of glial physiological functions are emerging as causally linked to brain diseases. In the healthy brain, astrocytes maintain ion homeostasis of the microenvironment, provide structural and metabolic support, regulate synaptic transmission, water transport and blood flow [9]. Additionally, microglia continuously extend and retract their process to interact with neurons and other types of glial cells, including astrocytes. Microglial phagocytosis (alongside other mechanisms, such as synaptic stripping and “trogocytosis”) plays an important role in the engulfment of synaptic elements [10–16]. Recent studies also revealed that astrocytes contribute to phagocytic clearance in a similar manner during normal physiological conditions [17] and there is abundant evidence that microglia and astrocytes communicate with each other [18–22]. It was further proposed that astrocytes can ingest aggregated proteins from the extracellular environment, suggesting that astrocytes keep, in coordination with microglia, the brain clean [23–26]. Since the elimination of unwanted and potentially harmful matter is crucial for central nervous system (CNS) function, dysregulation of glial phagocytosis and degradation might have a key role in PD pathogenesis. PD-related genes are expressed in astrocytes and microglia at levels comparable to neurons, while proteins encoded by several of these genes are implicated in degradative processes [27–30]. Here, we will discuss the role of glial phagocytic clearance in pathological conditions and the possible implication of PD-linked mutations in this important process. Finally, we will discuss the therapeutic potential of enhancing or restoring glial cell phagocytic and degradative capacity as future treatment for PD.

### Mechanisms of phagocytosis

**Fig. 1:**
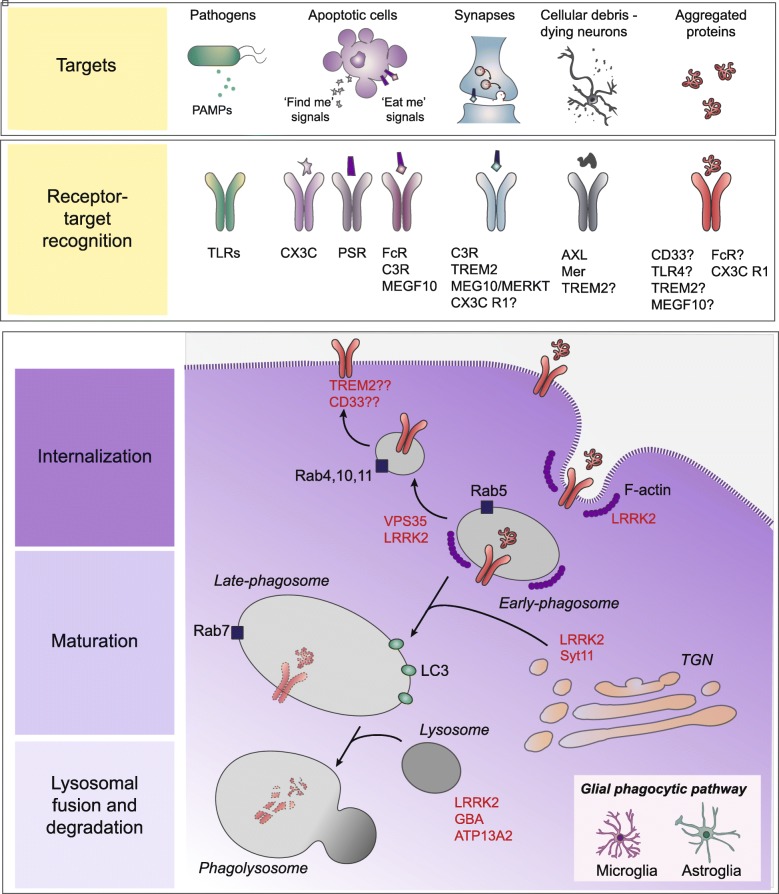
Phagocytic pathway in glial cells. Glial cell can recognize and uptake extracellular materials (targets) via specific receptors (receptor-target recognition). Targets are internalized and degraded through the endolysosomal machinery. Several PD-linked proteins intervene in the endolysosomal machinery and are highlighted in red

Phagocytosis is defined as the cellular uptake of particles (> 0.5 μm) within a plasma membrane envelope and partly overlaps with other cellular engulfing mechanisms such receptor-mediated endocytosis and macropinocytosis [31]. Phagocytes comprising monocytes, macrophages, dendritic cells, Langerhans cells, osteoclasts and microglia [32, 33], are responsible for the clearance of infectious agents, dead cells and tissue debris and are involved in the immune response. Astrocytes are also considered phagocytes and are able to ingest cellular debris and other material such as synaptic elements [34, 35]. During phagocytosis, self and non-self target particles are recognized by specific receptors on the plasma membrane and the target-receptor recognition depends on specific engulfment signals (Fig. 1, Targets; Receptor-Target recognition). Apoptotic cells secrete molecules called ‘find me’ signals to attract phagocytes toward them (e.g. lysophosphatidylcoline, LPC; sphingosine-1-phosphate, S1P; CX3C motif chemokine ligand 1, CX3CL1; nucleotides). For instance, secreted CX3CL1 binds to CX3C motif chemokine receptor 1 (CX3CR1) on microglia resulting in their migration [36]. Dying cells expose ‘eat me’ signals to be engulfed by macrophages, of which the best-characterized are the externalized phosphatidylserine (PS) on the cell surface. Exposed PS are recognized directly by PS receptors or indirectly by bridging molecules named opsonins (e.g. MFG-E8, Gas6, C3 and C1q). Fc receptor γ (FcRγ), the complement receptor 3 (CR3) and MEGF10, which are classified as opsonic receptors, are expressed in glial cells [37, 38]. FcRs bind to the constant (Fc portion) of immunoglobulin IgG or IgA antibodies whereas CRs bind to C3 deposited on the particle following complement activation. In microglia, pathogen-associated molecular patterns (PAMPs) are recognized by Toll-like receptors (TLRs) as well as by scavenger receptors (SRs), which are both non-opsonic receptors. Of note, TLRs are also expressed in astrocytes and TLR signaling can activate astrocytes to mediate neuroinflammation [39–42]. Upon recognition by the appropriated receptor, additional receptors are recruited eliciting complex signalling cascades that involve cytoskeletal rearrangement of actin filaments (F-actin) (see Fig. 1, Internalization). Actin polymerization is the force driving membrane extension and phagosome formation. Cdc42 and Rac were identified as players downstream of FcγR, while RhoA is implicated in phagocytosis mediated by CR3 [43]. One set of well characterized Cdc42/Rac effectors is the WASP family, WASP and WAVE, which are actin nucleation complexes that activate the Arp2/3 nucleation hotspot leading to the remodelling of actin network. In macrophages, uptake of exogenous particles and their degradation share features with autophagy, a process that incorporates endogenous organelles and misfolded proteins, and converges onto the lysosomal pathway (reviewed in [44, 45]) (see Fig. 1, Maturation). Specifically, maturating phagosomes acquire microtubule-associated protein 1A/1B-light chain 3 (LC3) and the recruitment of LC3 is mediated by the ubiquitin-like conjugation system proteins, Atg5 and Atg7 [46–48]. During LC3-associated phagocytosis (LAP), the phagosome surface decorated with phosphatidylinositol-3-phosphate (PI3P) binds to the Atg8/LC3 conjugation machinery, including Atg5, 12, and 16 L1, and NADPH oxidase 2 (NOX2)-dependent reactive oxygen species (ROS) production is required for LAP [49]. Atg8/LC3 conjugation to phagosomes regulates their fusion with lysosomes (see Fig. 1, Lysosomal fusion and degradation). Notably, no study has yet assessed whether LAP occurs in microglia and astrocytes, together with the consequences of LAP deficiency or dysfunction in the CNS. Nonetheless, given that glial cells are phagocytic, as notably shown in vivo, and that the proteins discussed above are expressed in both astrocytes and microglia, then it is reasonable to infer that these cells are capable of LAP. Once internalized, the phagosome can fuse selectively with early endosomes and acquire the small GTPases Rab5. The transition from an early phagosome to a late phagosome is marked by the conversion from Rab5 to Rab7. Rab7 activity is essential for the movement of phagosomes to the cell centre and, therefore, for further phagosome maturation [50]. In addition, the phagosome acquires lysosomal-associated protein 1 and 2 (LAMP1 and LAMP2), which are required for phagolysosomal fusion. During the maturation phase, other vesicles, from endoplasmic reticulum (ER) and *trans*-Golgi Network Golgi (TGN) complex, can be recruited to promote membrane extension [51]. Certain phagosomal cargo proteins are recycled back to the plasma membrane or the TGN, mediated by different Rab proteins (Rab4, Rab11, and Rab10) and the retromer complex, respectively. Overall, these observations demonstrate that the phagocytic pathway is highly dynamic, involves several protein components, and requires extensive membrane remodelling and recycling events that intersect with other cellular processes such as autophagy.

### Microglial phagocytosis and its role in PD

During development, microglia are specifically involved in synaptic pruning [52], notably by engulfing and eliminating viable C3/C1q-positive synaptic structures via C3R [53]. Microglia are also responsible for the elimination of apoptotic neurons generated in the context of adult neurogenesis in a process mediated by two TAM-receptor kinases, AXL and MER [54, 55]. However, microglia do not have a monopoly on synaptic pruning or CNS cleaning as astrocytes also play crucial roles in these processes (see next section). Recent data also indicate that microglial Triggering receptor expressed on myeloid cells 2 (TREM2) controls the process of synaptic pruning during postnatal development [56]. TREM2 is a cell surface receptor of the immunoglobulin superfamily that is expressed is several cell types including microglia. When activated, TREM2 signals promote survival, proliferation, secretion of cytokines, and, importantly, phagocytosis [57]. Therefore, the role of microglia in phagocytosis of synapses, when still viable, and neurons, when dead or dying, suggests that aberrations in these normal functions can contribute to synaptic loss and neurodegeneration. A clear link between impaired microglial phagocytosis and neurodegenerative diseases emerges from human genetic studies, including genome-wide association studies (GWAS). Indeed, variants in *TREM2* are well-known for increasing the risk for Alzheimer’s disease (AD), reviewed in [57]. Supporting the notion that deficient microglial phagocytosis might trigger neuronal death, several other genes with a role in amyloid β (Aβ) clearance were shown to confer susceptibility to AD (e.g. *CD33*, *CLU* and *CR1*) [58, 59]. Interestingly, *TREM2* and *CD33* variants have been associated with PD [60–62] and the recycling of microglial TREM2 at the plasma membrane was suggested to be regulated by the vacuolar protein sorting 35 (VPS35) [63], which is associated with autosomal dominant PD [64]. However, the genetic data linking TREM2 with PD have not been replicated [65] and the involvement of TREM2 in PD still requires investigation.

Analysis of histological samples from human PD brains revealed the presence of antigen presentation markers and a generally increased pro-inflammatory status [66–68] suggesting that microglial reactivity might compromise their homeostatic support to neurons, further influencing the pathogenesis of PD. Near the remaining nigral DA neurons, microglia also displayed morphologies characteristic of phagocytic cells, similar to those seen in aging [69]. However, the presence of reactive microglia alone does not define the helpful or harmful role that their phagocytosis plays in PD neuropathology.

Microglia show reactive phenotypes in animals injected with 6-hydroxydopamine (6-OHDA) or 1-methyl 4-phenyl 1,2,3,6-tetrahydro-pyridine (MPTP), two toxins widely used to produce lesions of DA neurons, and in neuron-glial co-cultures upon treatment with the pesticide rotenone [70–73]. Microglia-linked neuroinflammation was associated with an increased expression of microglial synaptic pruning receptor, CR3, in rotenone-exposed rats [74] and the presence of complement activation markers in human patients [75]. Of note, the knockout of C3, the component of complement system that induces phagocytosis through its interaction with C3R, rescues DA neurodegeneration in mice upon injection of the bacterial component lipopolysaccharide (LPS) [76]. Overall, these results suggest that DA loss might depend on pro-inflammatory microglia, exerted through an aberrant activation of the complement-phagosome pathway.

The involvement of microglial phagocytosis in PD pathogenesis is further supported by the fact that microglia uptake and remove DA cell debris in vivo. Moreover, microglia can also engulf α-syn, the deposited protein in LBs, possibly via the TLR4 receptor [72, 77, 78]. Considering the recently reported ability of misfolded α-syn released by degenerating neurons to propagate to other cells [79, 80], it is important to consider if glial-mediated phagocytosis plays a role in this spreading process. One possibility is that ineffective α-syn degradation in microglia may result in α-syn release through extracellular vesicles which in turn contributes to the diffusion of toxicity. Extracellular α-syn or neuronal debris might contribute to neurodegeneration by interfering with microglial phagocytic functions. For example, aggregated but not monomeric α-syn can inhibit phagocytosis by blocking FcγR signalling [78, 81]. Additionally, excess α-syn compromises phagocytosis as demonstrated by measuring fibrillar α-syn uptake in iPSC-derived macrophages from PD patients with α-syn (*SNCA*) A53T and *SNCA* triplication [82]. Consequently, extracellular α-syn may accumulate due to impairment of the microglial phagocytic molecular machinery.

Additional evidence suggests that several proteins encoded by genes implicated in mendelian forms of PD or positioned within PD risk *loci* impinge on microglial phagocytic activity. Leucine rich repeat kinase 2 (LRRK2) regulates the phagocytic response of myeloid cells via specific modulation of the actin-cytoskeletal regulator, WAVE2 [83]. Specifically, the G2019S mutation in LRRK2, a relatively common cause of familial PD, induces a WAVE2-mediated enhancement of phagocytic response in macrophages and microglia from patients and mouse models [83]. Consistent with this observation, LRRK2 depletion decreased microglial phagocytosis and inhibition of LRRK2 kinase activity reduced Tat-induced microglial phagocytosis in the BV2 immortalized microglial cell line [83, 84]. However, whether LRRK2-mediated increases of phagocytosis might contribute to DA-neuronal loss in animal models of PD has not been reported to date. Interestingly, LRRK2 is a negative regulator of *Mycobacterium tuberculosis* phagosome maturation in mouse and human macrophages [85]. This result suggests that LRRK2 may have evolved as part of the response to infectious diseases.

LRRK2 knockout mice also display reduced microglial expression of the fractalkine receptor CX3CR1 [86]. Signalling between the neuronal chemokine fractalkine (CX3CL1) and its receptor (CX3CR1) expressed by microglia, is a main mode of communication between neurons and microglia in the brain [36]. In CX3CR1 knockout mice, microglial phagocytosis was shown to be increased [87, 88]. However, the role of fractalkine signalling in the regulation of microglial phagocytosis still remains unclear.

Mutations in the *DJ-1* gene are known to be associated with rare cases of autosomal recessive PD [89]. DJ-1 acts as a multifunctional protein involved in gene transcription regulation and anti-oxidative defence. However, DJ-1 loss-of-function in microglia induces a reduced expression of TREM2 [90]. Further investigation demonstrated that DJ-1 knock-down reduces cell-surface lipid raft expression in microglia and impairs their ability to uptake and clear soluble a-syn, possibly by impacting on the autophagy pathway [91].

*Syt11*, the encoding gene for Synaptotagmin-11 (Syt11) is contained within a genome wide associated study-identified locus implicated in PD risk [92]. Syt11 localized to the TGN and recycling endosomes and appears to be involved in cytokine secretion and phagocytosis in microglia [93]. Syt11 was recruited to phagosomes and Syt11 deficiency inhibits microglial phagocytosis of α-syn fibrils, supporting its association with PD [93].

Additionally, in a mouse model that overexpresses human mutant α-syn in neurons of the spinal cord, microglial density and expression of AXL were increased [55]. As mentioned above, AXL, together with MER and TYRO3, is part of the TAM receptor family of tyrosine kinases regulating microglial phagocytosis of newborn apoptotic cells in the context of adult neurogenesis. In the α-syn transgenic mouse, loss of both MER and AXL increased the lifespan [55]. It was thus proposed that microglia could remove distressed motor neurons in this model through TAM receptor-mediated “phagoptosis” of living neurons [94]. In this case, microglial phagocytosis was not reduced, instead exacerbated and perhaps mistargeted, to living neurons, thus becoming pathological.

In conclusion, microglia can phagocytose dead and dying neurons, but also viable ones, as well as synapses and aggregated α-syn, which are all potentially relevant to PD pathogenesis. Therefore, a delicate regulation of the microglial phagocytosis seems to be critical in PD. On the one hand, elevated CNS inflammation might exacerbate microglial phagocytosis, causing enhanced, mistargeted, and potentially pathological clearance capacity. On the other hand, aging and/or genetic factors that alter substrate recognition, incorporation, sorting and lysosomal degradation might compromise microglial phagocytic clearance function, hence contributing to increased unwanted material among the CNS environment.

### Astrocytic phagocytic activity: does it contribute to PD development?

Astrocytes are the most abundant glial cells in the brain and have a range of essential functions required for maintaining neuronal health. Importantly, both microglia and astrocytes are inflammatory cells and many functions described for microglia are also shared by astrocytes. Specifically, astrocytes are crucial regulators of innate and adaptive immune responses in the injured CNS [95]. They respond to pathological states by secreting inflammatory mediators and increasing their expression of GFAP. However, in the context of PD, whether the effects of astrocyte activation plays a positive or negative role in disease outcome is still not well understood. As for microglia, it has been reported that astrocytes actively participate in phagocytic processes [96]. Consistent with this finding, transcriptome analysis identified high expression of genes involved in engulfment and phagocytosis in astrocytes [97]. However, the molecular mechanisms behind astrocytic pruning are still being elucidated. In 2013, Chung et al. reported that astrocytes phagocytize synapses in vitro and in vivo through a conserved pathway that involves two membrane receptors, MEGF10 and, as found for microglial cells, MER [98]. Once internalized, neuronal material is carried to lysosomes where degradative processes take place [98, 99]. MEGF10 also mediates the clearance of apoptotic cells in vitro and in mice, and the process appears to be mediated by binding of MEGF10 to the bridging molecule C1q associated with PS [100, 101]. MEGF10 is the orthologue of draper in *D. melanogaster* and is capable to intracellular signalling via specific downstream effectors (e.g. GULP1/ced-6) that control cytoskeletal rearrangements and lysosomal degradation [102, 103]. Of note, the stimulation of phagocytic activity by the overexpression of Draper/MEGF10 provides neuroprotection in an AD model of *Drosophila* reducing levels of Aβ peptides, reversing locomotor defects, and extending lifespan [104]. Similar to MEGF10, MER is highly expressed in astrocytes and recognizes specific bridging molecules (e.g. Gas6) that binds phophatidylserine in target debris [97, 105, 106].

Astrocytic phagocytic processes appear to be altered during prolonged stress, aging and diseases. Upon sleep deprivation in mice, the MER receptor is upregulated and astrocytic phagocytosis of presynaptic components is enhanced, suggesting a role in the recycling of heavily used synapses [17]. The involvement of MER in phagocytizing neuronal materials both in astrocytes and microglia might suggest overlapping functions between the two types of cells. In pathological conditions, reactive astrogliosis is characterized by a profound change in astrocyte phenotype in response to injury or disease [107]. An intimate crosstalk exists between microglia and astrocytes. By releasing signaling molecules, both microglia and astrocytes establish a reciprocal communication during neuroinflammation (reviewed in [21]). In this regard, it has been proposed that neuroinflammation associated with ischemia can induce two different types of reactive astrocytes named A1 and A2, respectively [19, 108]. A2 are protective and, possibly, promote CNS recovery and repair during ischemia [19]. For example, Morizawa et al. showed that astrocytes enhance phagocytosis, upregulate ABCA1, a protein known to be involved in cellular phagocytosis in the periphery, and its pathway molecules, MEG10 and GULP1, and contribute to the remodelling of damaged tissues after transient ischemic injury in vivo [34]. In contrast, A1 astrocytes are neurotoxic and lose many normal astrocytic functions including the ability to support synapse formation and function and instead tend to phagocytize neuronal elements. It has been demonstrated that activated microglia induce A2 to A1 phenotypic conversion of astrocytes by the release of Il-1, TNF and C1q [18]. Of note, the neurotoxic A1 astrocyte population is specifically observed in human neurodegenerative diseases including AD and PD, and during normal aging, suggesting that impaired astrocytic phagocytic clearance may contribute to increasing neuronal debris and abnormal protein accumulation, thus triggering neuroinflammation and neurodegeneration processes [18, 19]. Indeed, phagocytic activity of astrocytes appears to be important as a mechanism recruited for clearing damaged cells [35, 109]. In vitro, the release of cellular materials from laser-irradiated astrocytes or neurons induces a phagocytic response in nearby astrocytes [35]. In rats, synaptic debris produced by the degeneration of the striatal DA terminals upon 6-OHDA injection in lateral ventricles were found within specific structures named spheroids that were transferred to astrocytes [109]. Astrocytes can phagocytize large amounts of α-syn fibrils in vitro [25, 26]. α-syn is endogenously expressed at low levels in astrocytes [110, 111], but it can spread from neurons to glial cells and from one astrocyte to another astrocyte through the extracellular space, possibly inside of extracellular vesicles or via direct cell-to-cell transfer [23, 110, 112]. Astrocytes are able to degrade fibrillar α-syn as well as human α-syn purified from LBs both in vitro and in ex vivo brain slices, suggesting an active role for these cells in the clearance of α-syn deposits [110]. However, the majority of fibrillar α-syn ingested by cultured astrocytes is stored intracellularly rather than degraded. The accumulation of aggregated α-syn in astrocytes also disrupts their lysosomes, impacts mitochondria homeostasis and may induce cell-to-cell transfer of α-syn via tunneling nanotubes [23, 25, 26]. Similar findings were reported for Aβ fibrils [26]. Consistent with these results, there is evidence that cortical and striatal astrocytes from PD patients brains contain extensive α-syn deposits [7, 8, 113–115]. It is hypothesized that misfolded α-syn can spread from affected to unaffected regions of the CNS, leading to neurodegeneration and the progression of PD pathology (reviewed in [116]). Therefore, ineffective degradation of neuron-derived α-syn by astrocytes might contribute to PD pathogenesis and to intervene with the kinetics of astrocytic phagocytic capacity might be useful to slow down α-syn toxicity and stop its transmission.

Similar to microglia, astrocytes phagocytize and this activity is physiologically important. Although their role in neurodegenerative diseases and specifically in PD is still emerging, compelling evidence suggests that loss of proper astrocytic phagocytosis might contribute to neurodegenerative process. Unfortunately, mechanistic details on specific neuronal tags that signal for astrocytic degradation, astrocytic receptors involved in the recognition of aggregated α-syn as well as delivery to lysosomes are still unknown. Moreover, whether PD-linked mutations influence astrocytic phagocytosis remains undetermined.

### Dysfunction in the endolysosomal pathway: implication for glial phagocytic clearance

The identification of genes linked to familial forms of PD with pathological and clinical overlap with the sporadic syndrome has improved our understanding of pathogenic mechanisms involved in this disease [117]. Genetic studies provided some evidence that endo-lysosomal dysfunction may play an important role in the pathogenesis of PD. Among the several loci associated with PD, at least 11 are either directly or indirectly connected to the autophagy-lysosomal pathway [29]. Moreover, other protein products of PD loci can be placed into cell biological machineries that impinge on endosomal membrane trafficking [118]. Since phagocytic clearance relies on functional endocytosis, sorting, vesicle transport and compartment acidification as well as degradation, it is conceivable that variants or mutations in all of these proteins might be responsible for altering the ability of glial cells to remove degenerating synapses and release toxic/aggregated proteins. Although the majority of the reported PD-linked proteins are present at notable protein levels in glial cells, their biological function and the implication of their pathological mutations have been deeply studied in neurons yet rarely in microglia and astrocytes. In this section, we will discuss relevant PD genes focusing on their expression, localization, function and potential participation in taking up and degrading extracellular materials.

Two PD-associated proteins are specifically located at the lysosome and are essential for lysosomal functionality. ATPase type 13A2 (ATP13A2) is a protein involved in lysosomal cation homeostasis that is linked to autosomal recessive familial PD [119, 120]. ATP13A2 accumulates within LBs and ATP13A2 levels are decreased in DA nigral neurons from patients with sporadic PD [121] suggesting that ATP13A2 loss-of-function might contribute also to sporadic PD. ATP13A2 is a transmembrane protein localized both in lysosomes and late endosomes and dysfunction of ATP13A2 diminishes lysosomal degradation and autophagic flux [122–124], exosome externalization [125], and induces an accumulation of fragmented mitochondria [124, 126, 127]. Taken together, these findings point to a role of ATP13A2 in controlling the endolysosomal and autophagic pathways. Loss of ATP13A2 leads to lysosomal dysfunction and subsequent α-syn accumulation in cell cultures [128] while ATP13A2-null mice exhibit motor deficits, widespread gliosis, and lysosomal abnormalities [129]. PD-linked mutations in ATP13A2 lead to several lysosomal alterations in patient-derived fibroblasts, including impaired lysosomal acidification, decreased proteolytic processing of lysosomal enzymes, reduced degradation of lysosomal substrates, and diminished lysosomal-mediated clearance of autophagosomes [122]. Similar to other proteins involved in PD, ATP13A2 is expressed by microglia [27] and astrocytes [28] and deficiency of ATP13A2 induces astrocyte-related intense inflammation which exacerbates DA neuron damage in co-cultures [130]. However, the precise role of ATP13A2 in the lysosomal pathway of glial cells and the impact of ATP13A2 loss-of-function in glial degradative capacity is as yet unexplored.

Mutations in *Glucocerebrosidase* (*GBA)* gene, which codes for a lysosomal enzyme implicated in a lysosomal storage disorder (LSD) called Gaucher’s disease (GD) in the homozygous state, are common risk factors for PD [131]. Clinically, PD patients with GBA mutations exhibit typical manifestations of PD or dementia with Lewy bodies. The glucocerebrosidase enzyme (GCase), which catalyzes the breakdown of sphingolipid glucosylceramide to ceramide and glucose within lysosomes and reduces the enzymatic activity caused by mutations, may lead to impaired lysosomal protein degradation and increased exosomal release of α-syn [132, 133]. Both GD and PD patients with GBA mutations show two common features: abnormal α-syn accumulation and glial cells reactivity, suggesting that gliosis may be an important contributor to pathogenesis [134]. Consistent with this idea, studies in Gba-deficient mice revealed that reactive microglia and astrocytes accumulate in degenerating brain regions [135]. Additionally, primary macrophages from patients with GD recognize and take up apoptotic cells but do not correctly digest engulfed cells [136]. This is caused by aberrant recruitment of phagosome-associated proteins, leading to substantially impaired phagosome maturation and phagosome-lysosome fusion [136]. However, only one report specifically analyzed autophagic and proteasomal machinery in mouse astrocytes lacking Gba showing that mitophagy was impaired [137].

As mentioned above, mutations in LRRK2 are a common cause of familial and sporadic PD [138–140]. LRRK2 plays a major role in vesicular membranes, as well as autophagy and lysosome biology [141], and its function is mediated by the phosphorylation of a subset of Rab GTPases (e.g. Rab8A and Rab10) [142, 143]. LRRK2 has been implicated in the regulation of lysosomal morphology and function in several models, including astrocytes [144–148]. Lysosomal abnormalities in different tissues have been observed upon the inhibition of LRRK2 kinase activity and in many LRRK2 knockout models [145–149]. iPSC-derived DA neurons from G2019S mutation carriers show an increase in autophagic vacuoles and an accumulation of aggregated α-syn [150]. In addition, vesicles with multiple membranes resembling autophagosomes or autophagic vacuoles within regions enriched in axons and/or synapses have been reported in transgenic mice overexpressing the G2019S LRRK2 mutation [151]. G2019S knock-in mutation in LRRK2 induces significant changes in lysosomal morphology and acidification, and decreased basal autophagic flux in mouse brain neurons. These changes are associated with an accumulation of detergent-insoluble α-syn and increased neuronal release of α-syn [152].

Similar to ATP13A2 and GBA, LRRK2 is highly expressed in mouse and human astrocytes and plays a key role in the lysosomal function of these cells [27, 144, 153]. In mouse primary astrocytes, LRRK2 co-localizes with the lysosomal markers, LAMP1 and LAMP2, and LRRK2 deficiency significantly enhances lysosomal number [144]. Interestingly, pathogenic mutations in LRRK2 in astrocytes produce enlarged lysosomes with a decreased degradative capacity and this effect is dependent on the kinase activity [144]. Thus, LRRK2-dependent lysosomal dysfunction in astrocytes might exacerbate the neurodegeneration process because of their inability to clear up unhealthy neurons and the released α-syn. In neurons, LRRK2 is also recruited to the TGN and promotes the clearance of Golgi-derived vesicles by interacting with and phosphorylating Rab7L1 (Rab29) [154–156]. Along this line, LRRK2 was shown to regulate late endocytic membrane trafficking in a Rab7-dependent manner while mutated LRRK2 delays the recycling of the epidermal growth factor receptor (EGFR) [157]. In addition, several papers point to a functional interaction between LRRK2 and VPS35, a core component of the retromer cargo recognition complex that plays a critical role in cargo retrieving pathway from the endosome to the TGN, involved in genetic PD [158–160]. PD-associated defects in LRRK2 or LRRK2 deficiency disrupt retromer cargo mannose 6-phosphate receptor (M6PR) trafficking and impairs secretory autophagy and Golgi-derived vesicle secretion [154, 161]. Expression of wild-type VPS35, but not a familial PD-associated mutant form (D620N), rescued these defects in *D. melanogaster* models [154]. Noteworthy, VPS35 D620N knock-in mutation strikingly elevates LRRK2-mediated phosphorylation of Rab8A, Rab10, and Rab12 in mouse indicating that the two proteins function in a converging pathway and that mutant VPS35 exacerbates LRRK2 toxicity [158]. Taking together, these observations might suggest possible implications of mutated LRRK2 and VPS35 in phagosome formation and/or phagocytic receptors internalization and recycling.

A recent characterization of the biological function of Transmembrane protein 175 (TMEM175), encoded by a gene located under one of the most significant PD GWAS peak [92], Biorxiv: 10.1101/388165, suggests a role in the pathogenesis of PD. Specifically, TMEM175 is a lysosomal K^+^ channel that stabilizes lysosomal pH and regulates lysosome catalytic activity [162–164]. In neuronal systems, TMEM175 deficiency decreases GBA activity and the lysosomal-mediated clearance of autophagosomes with a consequent impairment in the clearance of exogenous fibrillar α-syn [163]. Of note, TMEM175 mRNA was detected in brain glial cells with a particularly elevated expression in mouse and human astrocytes [27]. However, TMEM175 function is poorly understood and no data is available regarding the effect of its ablation in glial cells. Since lysosomal degradation serves as a key final step to resolve protein aggregation upon glial phagocytosis, it is likely that TMEM175 dysfunction negatively impacts on this pathway.

Concluding, a better understanding of the involvement of endo-lysosomal pathway and the implication of PD pathological mutations in the context of glial phagocytosis might be helpful to understand the molecular basis of neurodegeneration in PD.

### Conclusions and future directions

**Fig. 2:**
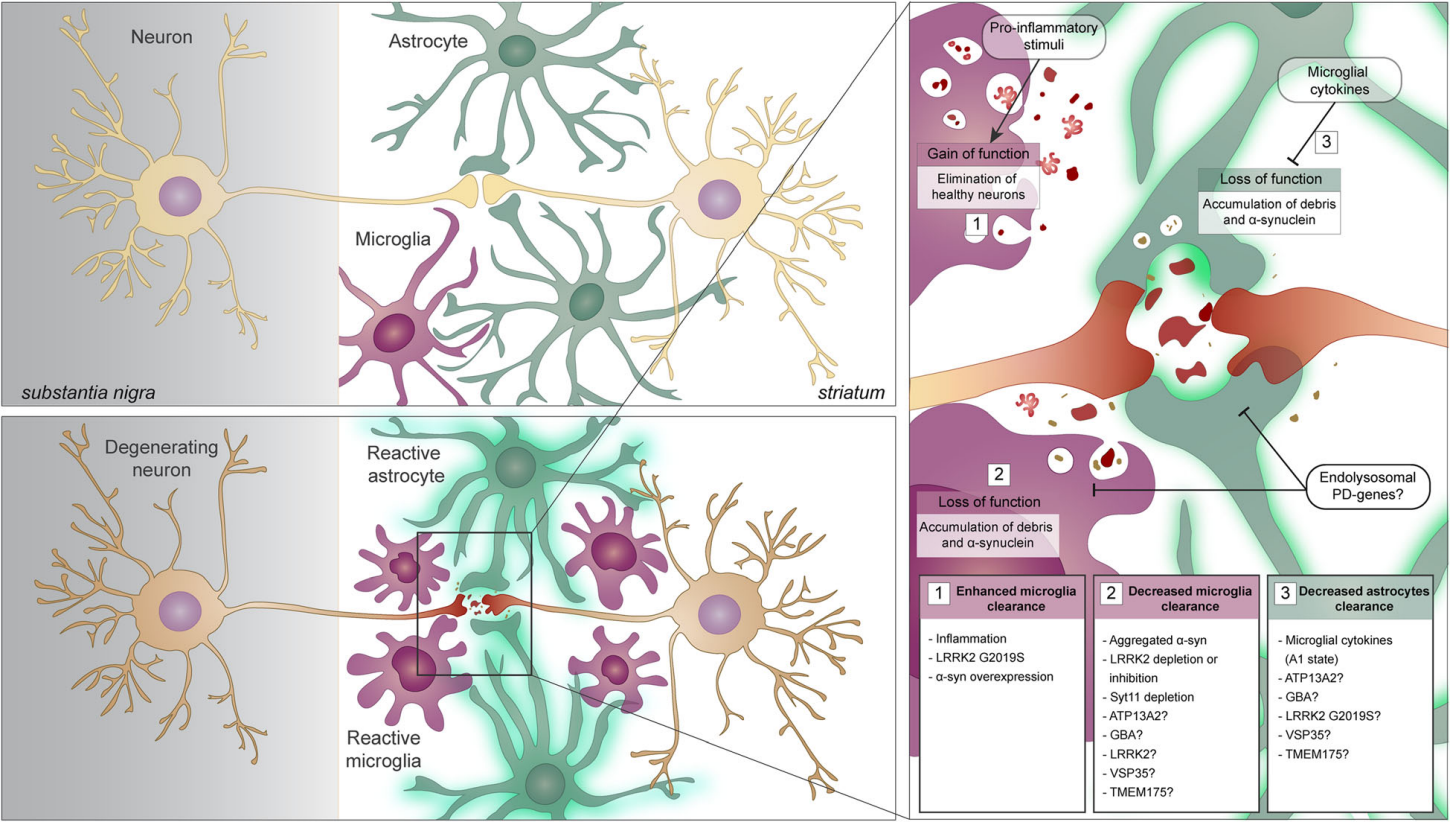
Phagocytic clearance dysfunction in PD neurodegeneration. **a** Dopaminergic terminals in the striatum are surrounded by astrocytic and microglial processes. **b** PD is characterized by dopaminergic neuronal death, proteinaceous accumulations positive for α-syn in the surviving neurons as well as in astrocytes, accompanied by extended gliosis and neuroinflammation. Reactive glia can phagocytize neuronal debris and released aggregated α-syn, attenuating neurodegeneration. Multiple factors can cause phagocytic clearance mistargeting or dysfunction (enhance or decrease) thus exacerbating the neurodegenerative process

The elimination of unwanted and harmful material is crucial for CNS homeostasis and the ability of certain brain cells to phagocytize is particularly relevant during neurodegeneration when neuronal debris and neuronal-released toxic proteins accumulate. Astrocytes surround neurons and closely encapsulate synapses and they might be required to constantly and promptly phagocytize and eliminate degenerating synapses and cell debris (Fig. 2a). Once the degenerating process becomes acute, microglia are recruited and start to exert their neuroprotective function by massively finalizing the clearance of cells and unfolded proteins. Exacerbated microglia-mediated neuroinflammation might be detrimental for more than a reason. First, enhanced microglial phagocytic activity might start to eliminate healthy synapses and second, the released proinflammatory cytokines might speed down astrocytic phagocytosis. Both PD causal genes and PD genetic risk factors involved in endosome maturation as well as lysosomal function encoded for proteins highly expressed in glial cells. In this scenario, glial cells carrying PD mutations could fail to degrade engulfed materials because of endo-lysosomal impairment. Since both microglia and astrocytes endogenously express low levels of aggregate-prone proteins including α-syn, the intracellular accumulation of undigested material could interfere with the glial homeostatic functions, thus affecting neuronal health and sustaining neurodegeneration (Fig. 2b).

Based on these considerations, could the modulation of glial phagocytic clearance serve as a valuable therapeutic approach in PD? Ideally, cell-targeted factors promoting lysosome acidification and/or biogenesis might enhance lysosomal function, thus allowing for improved degradative capacity. However, scarce information is available regarding the molecular mechanisms and the specific, possibly distinct, roles of microglial versus astrocytic phagocytic clearance in the onset and progression of PD. Future studies comparing the kinetics of microglia and astrocyte phagocytic activities are warranted to clearly dissect the molecular machineries as well as unravel the biological basis of communication between these two cell types in the CNS.

## Abbreviations

6-OHDA: 6-hydroxydopamine
AD: Alzheimer’s disease
CNS: Central nervous system
CR3: Complement receptor 3
CX3CL1: CX3C motif chemokine ligand 1
CX3CR1: CX3C motif chemokine receptor 1
DA: Dopaminergic
ER: Endoplasmic reticulum
F-actin: Actin filaments
FcRγ: Fc receptor γ
GBA: Glucocerebrosidase
GCase: Glucocerebrosidase enzyme
GFAP: Glial fibrillary acidic protein
GWAS: Genome-wide association study
iNOS: Inducible oxide synthase
LAMP1 and LAMP2: Lysosomal-associated protein 1 and 2
LAP: LC3-associated phagocytosis
LBs: Lewy bodies
LC3: Microtubule-associated protein 1A/1B-light chain 3
LNs: Lewy neurites
LPC: Lysophosphatidylcoline
LPS: Lipopolysaccharide
LRRK2: Leucine rich repeat kinase 2
M6PR: Mannose 6-phosphate receptor
MHC: Major histocompatibility complex
MPTP: 1-methyl 4-phenyl 1,2,3,6-tetrahydro-pyridine
NOX2: NADPH oxidase 2
PAMPs: Pathogen-associated molecular patterns
PD: Parkinson’s disease
PS: Phosphatidylserine
ROS: Reactive oxygen species
S1P: Sphingosine-1-phosphate
SRs: Scavenger receptors
Syt11: Synaptotagmin-11
TAM receptors: TYRO3, AXL, MER tyrosine kinase receptor
TGN: *Trans*-Golgi Network Golgi
TLRs: Toll-like receptors
TMEM175: Transmembran protein 175
TREM2: Triggering receptor expressed on myeloid cells 2
VPS35: Vacuolar protein sorting 35
α-syn: α-synuclein
